# Gaussian white noise stimulation as an alternative method to excite sensory neurons

**DOI:** 10.3389/fphar.2025.1561905

**Published:** 2025-04-22

**Authors:** Thomas Losgott, Klaus W. Schicker, Karlheinz Hilber, Stefan Boehm, Isabella Salzer

**Affiliations:** Division of Neurophysiology and Neuropharmacology, Center for Physiology and Pharmacology, Medical University of Vienna, Vienna, Austria

**Keywords:** Gaussian white noise, neuronal firing, dorsal root ganglion (DRG), dissociated cultures, inflammatory soup, NAPQI, retigabine

## Abstract

**Introduction:**

Peripheral nerve endings of dorsal root ganglion (DRG) neurons act as nociceptors and generate action potentials in response to noxious stimuli. Primary cultures of dissociated DRG have been used extensively to study changes neuronal excitability caused by either analgesics or pathological conditions, such as inflammation. The dissociation procedure can be viewed as a form of axotomy, and one might expect a resulting increase in excitability of the neurons during the subsequent culture period. However, changes in firing properties of DRG neurons over time *in vitro* have not been investigated systematically.

**Methods:**

Thus, the current experiments compared action potential firing in dissociated DRG neurons after one to 7 days in culture and examined Gaussian white noise as novel stimulation paradigm. Primary cultures of DRG neurons were recorded in perforated patch current-clamp. Action potentials were evoked either by a sequence of five rectangular current pulses with increasing amplitudes or by Gaussian white noise of varying RMS amplitudes and frequencies.

**Results:**

Conventional rectangular current injections triggered 19 ± 20 action potentials in cells when recorded within 24 h after dissociation. After 7 days in culture, DRG neurons fired 4.3 ± 0.7 action potentials in response to current pulses. Inflammatory mediators increased numbers of action potentials evoked by rectangular current pulses within 24 h after dissociation to 66 ± 54, but left those elicited after 7 days *in vitro* unaltered (4.3 ± 0.5). In the same set of neurons kept in culture for 7 days, Gaussian white noise stimuli triggered 1,540 ± 470 action potentials, and this number was increased to 2089 ± 685 by inflammatory mediators. The Kv7 channel activator retigabine and the paracetamol metabolite n-acetyl-p-benzoquinone imine (NAPQI) decreased numbers of action potentials triggered by Gaussian white noise, but failed to do so when rectangular current pulses were used as stimuli, both in neurons after 7 days in culture.

**Discussion:**

These results demonstrate a decrease in the excitability of DRG neurons from day one to 7 after dissociation and reveal Gaussian white noise as reliable trigger of action potential firing in these neurons.

## 1 Introduction

The sensation of pain, with the exception of central neuropathic pain, starts at the level of nociceptive neurons. The peripheral nerve endings of these act as nociceptors, i.e., sensors that are able to transduce noxious stimuli into generator potentials which are then transformed into trains of action potentials (Loeser and Treede, 2008). In general, the more action potentials are generated in peripheral nociceptive neurons the more intense the accompanying pain sensation will be, and this holds true for nociceptive as well as inflammatory or neuropathic pain (Middleton et al., 2021). The somata of these neurons are located in trigeminal or dorsal root ganglia (DRG) and are thus easily accessible. Accordingly, numerous publications have investigated action potential firing in neurons of these ganglia *in vitro*, be it within intact ganglia or in dissociated primary cultures (Körner and Lampert, 2022).

Dissociated primary cultures of sensory ganglia are extremely versatile experimental models, as they do not only offer direct access for patch-clamp investigations of ion channels that control the firing of action potentials and thus neuronal excitability (Körner and Lampert, 2022). They can even be used to mimic pathologic conditions that lead to chronic forms of pain, such as inflammatory or neuropathic pain. With respect to the former, cultures can be exposed to algogenic mediators that are collectively known as inflammatory soup (i.s.; Basbaum et al. (2009). Whether bradykinin (Liu et al., 2010), ATP, or other nucleotides (Yousuf et al., 2011), or serotonin (Salzer et al., 2016) are used, in their presence, DRG neurons fire many more action potentials than in their absence. With respect to neuropathic pain, cultures can be treated, for instance, with the anticancer agent paclitaxel, which causes chemotherapy-induced peripheral neuropathy in human patients and rats (Polomano et al., 2001). This induces a neuropathic phenotype in DRG cultures that is characterized by enhanced neuronal excitability (Li et al., 2021). While such algogenic conditions boost excitability of nociceptive neurons, analgesics lead to the opposite phenomenon. For example, gabapentin, a standard drug in the treatment of neuropathic pain, reduces action potential firing in DRG neurons (Yang et al., 2009). Likewise, N-acetyl-p-benzoquinone imine, an active metabolite of the widely used analgesic paracetamol, has been reported to diminish excitability in such neurons (Ray et al., 2019). Most recently, a high throughput screening platform for the identification of novel analgesics has been devised that is based on optical recordings of action potentials in primary cultures of rat DRG (Liu et al., 2024).

During the preparation of dissociated primary DRG cultures, neuronal cell bodies are separated quite harshly from the axon. Accordingly, the dissociation procedure has been viewed as a model of axotomy, and dissociated DRG cultures were used to investigate consequences of this type of nerve injury. Along this line, muscle lim protein is induced in L4/L5 DRGs after sciatic nerve crush and its expression sharply increases in cultures of DRGs from day 1–5 after dissociation (Levin et al., 2017). Similarly, GTP cyclohydrolase 1 is induced in DRG neurons after nerve injury and in cultures from day 1–4 post dissociation (Cronin et al., 2022). The latter enzyme is rate-limiting in the *de novo* synthesis of tetrahydrobiopterin which contributes to neuropathic pain. Accordingly, one would expect that the time-dependent rise in protein expression would be paralleled by an increment in membrane excitability when dissociated DRG neurons are kept in culture for increasing periods of time, but this has not been tested (Cronin et al., 2022; Levin et al., 2017).

In principle, action potential firing in DRG neurons is characterized as either phasic or tonic: phasic firing means that only a single or a few action potentials (<4) can be seen at the beginning of a sustained injection (>0.5 s) of depolarizing currents, whereas >5 action potentials are evenly distributed throughout current injection during tonic firing. Using this categorization, several phenotypic features of DRG neurons, such as isolectin B4 positivity (Choi et al., 2007) or the expression of markers of nociceptive function (Viatchenko-Karpinski and Gu, 2016), have been found to correlate with the proportion of neurons that fire in either a phasic or a tonic manner. In addition, the firing properties have been assessed either one or 3–5 days after dissociation, and the percentage of neurons firing tonically was found to be 30 to 40 after one (Ditting et al., 2009; 2016; Lale et al., 2023) vs. <20 after 3–5 days (Sculptoreanu and de Groat, 2007; Sculptoreanu et al., 2009) in culture. Thus, excitability of DRG neurons appears to decrease rather than increase the longer the neurons are kept in culture after dissociation.

To clarify this issue, the current experiments compared firing properties of DRG neurons one, 3 to 4, and 7–10 days after dissociation. After 1 week, virtually none of the neurons investigated was able to fire more than one single action potential during prolonged current injections, and this firing pattern was not altered by inflammatory soup constituents. To reveal whether this apparent limitation of current pulse injections might be overcome by an alternative method of eliciting action potential firing in sensory neurons, Gaussian white noise stimulation was examined. This method has the advantage of delivering wave forms in random order without prejudgment as to their efficacies of triggering action potentials and it has been reported to reliably elicit firing in *Aplysia* neurons (Bryant and Segundo, 1976) and squid giant axons (Guttman et al., 1974). The results reveal that white noise stimulation is able to generate action potentials at constant frequencies in DRG neurons after 7 days *in vitro* (DIV) that otherwise produce only a single action potential in response to steady current injections, and this white noise-induced firing is modulated under pro-as well as analgesic conditions.

## 2 Materials and methods

### 2.1 Materials

Dimethyl sulfoxide (DMSO), amiloride, retigabine and bulk chemicals for electrophysiological solutions (see 2.3 Electrophysiology) were ordered from Sigma-Aldrich (Vienna, Austria). Retigabine stock solutions of 10 mM in DMSO were prepared and further diluted with external solution to a final concentration of 10 μ M, which resulted in a DMSO concentration of 0.1%. Amiloride was dissolved in DMSO, a 30 mM stock was stored at −20 ° C. Amphotericin B was bought from AppliChem GmbH (Darmstadt, Germany), a fresh stock solution of 50 mg ${\text{ml}}^{-1}$ was prepared daily and kept in the dark at room temperature. N-acetyl-p-benzoquinone imine (NAPQI) was custom synthesized by abcr (Karlsruhe, Germany), a stock solution of 10 mM in DMSO was prepared and stored at −80 ° C. Every day, NAPQI was freshly diluted to 3 mM in DMSO and stored at −20 ° C for the rest of the day. Once every 2 h, a fresh external solution containing NAPQI was prepared. Adenosine diphosphate (ADP) disodium salt, adenosine triphosphate (ATP) disodium salt, bradykinin acetate, histamine dihydro chloride, and serotonin hydrochloride were purchased from Sigma-Aldrich (Vienna, Austria). Prostaglandin ${\text{E}}_{2}$ ( ${\text{PGE}}_{2}$ ) was from MedChemExpress LLC (Monmouth Junction, NJ, United States; distributed by THP Medical Products, Vienna, Austria), the PAR2 agonist 2-furoyl-LIGRLO-amide was purchased from TargetMol (Wellesley Hills, MA, United States; distributed by Eubio, Vienna, Austria), substance P was from HelloBio (Bristol, United Kingdom). ADP (10 mM), ATP (100 mM), bradykinin (1 mM), histamine (100 mM), serotonin (10 mM), and substance P (10 mM) were dissolved in deionised water, and ${\text{PGE}}_{2}$ (10 mM) was dissolved in DMSO, which were all further diluted in deionised water before being added to the external buffer. A stock solution of 2-furoyl-LIGRLO-amide (10 mM) was prepared in DMSO and stored at −80 ° C. Before use, it was further diluted to 1 mM in DMSO and stored at −20 ° C. Capsazepine was from Cayman Chemicals (Ann Arbor, MI, United States; distributed by Szabo-Scandic, Vienna, Austria) and dissolved in DMSO (10 mM). Capsaicin was purchased from Chemodex (St. Gallen, Switzerland; distributed by Eubio, Vienna, Austria). A stock solution (100 mM) was prepared in NaOH and diluted to 300 μ M in deionized water before use. Tetrodotoxin (TTX) was purchased from Latoxan (Valence, France) and dissolved in acetic acid (1 mM) and stored at +4 ° C.

### 2.2 Primary cultures of rat dorsal root ganglion (DRG) neurons and viral transduction

Primary cultures of DRG neurons were prepared as described before (Ray et al., 2019) in full accordance with the Austrian animal protection law and animal experiment by-laws. Sprague-Dawley rat pups (RRID: MGI:5651135) of both sexes were decapitated 10–15 days after birth. Dissection of dorsal root ganglia was achieved by opening the spine with two longitudinal cuts in the sagittal plane through the vertebral bodies (ventral) and arches (dorsal) and then moving the spinal cord to the side. After isolation, the ganglia were incubated in a collagenase (1.5 mg ${\text{ml}}^{-1}$ ; Sigma-Aldrich, Vienna, Austria) and dispase (3.0 mg ${\text{ml}}^{-1}$ ; Roche, Vienna, Austria) solution for 30 min at 37 ° C and, afterwards, trypsinized (0.25% trypsin, Worthington, Lakewood, NJ, United States) for 10 min at 37 ° C. Subsequently, the ganglia were washed twice with 2 mL ${\text{Ca}}^{2+}$ -free Tyrode solution, which contained (in mM) NaCl (150), KCl (4), ${\text{MgCl}}_{2}$ (2), glucose (10), and HEPES (10) (pH 7.4 adjusted with NaOH). The cells were dissociated by trituration with fire polished glass Pasteur pipettes and resuspended in Dulbecco’s Modified Eagle Medium (DMEM; Sigma-Aldrich, Vienna, Austria) containing 10 mg ${\text{l}}^{-1}$ insulin (Sigma-Aldrich, Vienna, Austria), 50 μ g ${\text{l}}^{-1}$ nerve growth factor (NGF; R&D Systems Minneapolis MN, United States), 25,000 IU ${\text{l}}^{-1}$ penicillin (Sigma-Aldrich, Vienna, Austria), and 25 mg ${\text{l}}^{-1}$ streptomycin (Sigma-Aldrich, Vienna, Austria). Finally, for electrophysiological recordings, 50,000 cells were seeded into glass rings with an inner diameter of 6.5 mm, which had been placed in 35 mm culture dishes (Nunclon delta-surface, ThermoFisher scientific, Waltham MA, United States) coated with poly-D-lysine (Sigma-Aldrich, Vienna, Austria). Two hours after seeding, the glass rings were removed from the culture dishes, and the medium was supplemented with 5% heat-inactivated fetal bovine serum (FBS-hi; Biowest, Nuaille, France). On the day after preparation, the medium was exchanged with DMEM plus insulin, NGF, penicillin, streptomycin, (see above), and 5% FBS-hi. The cultures were kept at 37 ° C in a humidified ${\text{CO}}_{2}$ (5%) atmosphere for up to 24 h (DIV 1), 3–4 days (DIV 3), or 7–10 days (DIV 7) before being used for electrophysiological recordings (preliminary experiments did not reveal any differences between DIV 7 and DIV 10 neurons). For ${\text{Ca}}^{2+}$ imaging experiments, 50,000 cells were seeded onto poly-D-lysine coated glass-bottom dishes with a diameter of 35 mm (P35G-1.5–10-C, MatTek, Ashland, MA, United States). One day after preparation, the culture medium was replaced with one constisting of DMEM, plus insulin, NGF, penicillin, streptomycin, and 5% FBS-hi, as well as an adeno-associated viral (AAV) construct containing hSYN-jGCamP8s (Losgott et al., 2025). Imaging experiments were performed after 7 days in culture.

### 2.3 Electrophysiology

Current-clamp recordings were conducted at room temperature (20–24 ° C) using an Axopatch 200B amplifier (RRID: SCR_018866), a Digidata 1440 interface (RRID: SCR_021038), and the Clampex 10.7 software (Molecular Devices, San Jose, CA, United States, RRID: SCR_011323). All electrophysiological measurements were carried out using the perforated patch-clamp technique. Borosilicate glass capillaries (GB150-8P, Science Products, Hofheim, Germany) were used to manufacture patch pipettes with a Sutter P-97 puller (Sutter Instruments, Novato, CA, United States, Sutter, RRID: SCR_018636). Pipette tip resistances were in the range of 1.5–4.5 M Ω . The patch pipettes were front-filled with an internal solution containing (in mM) ${\text{K}}_{2}{\text{SO}}_{4}$ (75), KCl (55), HEPES (10), and ${\text{MgCl}}_{2}$ (8), adjusted to pH 7.4 using KOH, and then, back-filled with the same solution containing 750 μ g/mL amphotericin B (1.5% DMSO). Recordings were started after >30 min when the series resistance had stabilized and remained below 20 M Ω . The external solution contained (in mM) NaCl (140), glucose (20), HEPES (10), ${\text{CaCl}}_{2}$ (2.5), ${\text{MgCl}}_{2}$ (2), KOH (3), and NaOH (2), with a pH of 7.4. The liquid junction potential between internal and external solution was calculated with the “Calculate Junction Potentials” tool of the Clampex 10.7 software to amount to 8 mV and was corrected for during the measurements. To activate transient receptor potential vanilloid 1 (TRPV1) or acid-sensing ion channels (ASIC), a low pH external solution was used, which contained (in mM) NaCl (140), glucose (20), 2-(N-morpholino)ethanesulfonic acid (MES) (10), ${\text{CaCl}}_{2}$ (2.5), ${\text{MgCl}}_{2}$ (2), KCl (3), NaOH (2), and resulted in a pH of 5.5. During electrophysiological recordings, the cells were perfused using an Octaflow perfusion system (ALA Scientific Instruments Inc., Clarksburg, NJ, United States), which allows for solution exchange around the cell within 50–150 ms. All electrophysiological data were analyzed with the Clampfit 10.7 software (Molecular Devices, San Jose, CA, United States).

To test for TRPV1- or ASIC-positive DRG neurons, non-spontaneously firing small- or medium-sized cells (membrane capacitance <30 pF) were clamped to a membrane potential of −70 mV and, every 3 min, superfused with a low pH external solution for 15 s, which elicited an inward current in the majority of cells. If this current was blocked by 10 μ M capsazepine, the cells were considered TRPV1-positive. Similarly, if the current was inhibited by 30 μ M amiloride, the neurons were classified as ASIC-positive. Only TRPV1- and/or ASIC-positive cells were used for excitability measurements. No further subclassification of neurons was conducted. Currents were low-pass filtered at 2 kHz and recorded with a sampling rate of 10 kHz. For recordings using current pulse injections, the rheobases of each DRG neuron was determined by injecting 2-s current pulses, starting with 5 pA, and increasing current amplitudes by 5 pA for DIV 1 neurons, and 10 pA with 10 pA increments for DIV 3 and DIV 7 neurons. Afterwards, action potential firing was triggered by injecting five 2-s rectangular pulses of increasing intensities starting from 1 x, 1.5 x, 2 x, 2.5 x, to 3 x rheobases. Subsequently, the neurons were stimulated with filtered Gaussian white noise. The protocol included 35 consecutive 10-s stimuli with filter frequencies of 200, 100, 50, 25, and 12.5 Hz (4-pole digital Bessel zero phase filter) and increasing root mean square (RMS) amplitudes of 50, 100, 150, 200, 250, 300, and 350 pA. A second, shorter, white noise stimulus protocol was created for time course recordings (Figure 5) consisting of 25 consecutive 2.5-s stimuli with filter frequencies of 200, 100, 50, 25, and 12.5 Hz (4-pole digital Bessel zero phase filter) and increasing RMS amplitudes of 100, 150, 200, 250, and 300 pA. The white noise stimulation files were created with MatLab (MATLAB version R2022a, The MathWorks Inc., Natick, MA, United States, RRID: SCR_001622) using the source code provided as (Supplementary material).

DRG neuron excitability was first recorded in solvent (DMSO 0.1%) followed by perfusion with an artificial inflammatory soup consisting of ADP (1 μ M), ATP (1 μ M), bradykinin (30 nM), histamine (1 μ M), the PAR2 agonist 2-furoyl-LIGRLO-amide (1 μ M), ${\text{PGE}}_{2}$ (300 nM), serotonin (300 nM), and substance P (10 nM); or TTX (1 μ M). For time course recordings (Figure 5), action potentials were alternatingly triggered by the short white noise stimulus protocol immediately followed by a conventional 5-step pulse protocol. A baseline was recorded in presence of solvent (DMSO 0.1%) consisting of two noise and two pulse stimulus protocols, before either retigabine (10 μ M) was applied for 300 s, followed by a wash-out with solvent, or NAPQI (3 μ M) was applied for 600 s (Figure 5). For a direct comparison between two stimulation methods, numbers of action potentials triggered by a single set of white noise stimuli and current pulses, respectively, were normalized to an average of the first two in a series of 9 stimuli.

### 2.4 Calcium imaging

Calcium imaging experiments were conducted on a Nikon Eclipse Ti2 microscope with a 40 × 1.25 NA water immersion objective (Nikon, CEE, Vienna, Austria). Neurons expressing jGCamP-8s were excited at 490 nm (pE4000 CoolLed, Andover, United Kingdom) and emission was read out using a GFP filter (excitation filter: 470/40 nm, dichroic: 500 nm, emission filter: 535/50 nm; Semrock, NY, United States). Image acquisition was conducted using an electron-multiplying charge-coupled device (EMCCD) camera (iXon 888 Ultra, Oxford Instruments, Oxford, United Kingdom) in 16-bit mode, at 10-MHz readout speed, EM Gain of 40, and an exposure time of 20 ms, recording one frame every 1 s. Solutions were applied using an Octaflow 2 perfusion system (ALA Scientific Instruments Inc., Clarksburg, NJ, United States), which allows for solution exchange around the cell within 50–150 ms. Cells were perfused using the same external solution as in patch clamp experiments. Calcium transients were triggered during two 20 s periods, and the addition of either 20 mM KCl or 300 nM capsaicin to the external solution was used as stimulation paradigm. Image series were analyzed with ImageJ (Schindelin et al., 2012). First, images were background corrected by subtracting the average intensity of a cell-free region of interest for each image. Δ ${F/F}_{0}$ was calculated by subtracting the average fluorescence intensity value of the first 5 images ( ${\text{F}}_{0}$ ) from the region of interest (ROI) intensity (F), and normalizing it to ${\text{F}}_{0}$ (Equation 1):

$$Y=\frac{F-F_{0}}{F_{0}}$$ (1)

### 2.5 Data analysis and statistics

For current pulse injections, action potentials were counted with the “Threshold Search” function of the Clampfit 10.7 software by setting a threshold value, which was in the range of 30–90 mV more positive to the resting membrane potential. Signals that exceeded that value were regarded as action potentials. The threshold value was chosen individually for each neuron depending on the amplitude of the stimulation pulse. For the analysis of white noise stimulation recordings, traces were first filtered with an 8-pole 600 Hz high-pass Bessel filter to remove the noise. The remaining signals, which corresponded to action potentials, were then detected and counted using the “Template Search” function of the Clampfit 10.7 software (Figure 4A).

Data are shown as arithmetic means ± standard deviation (S. D.) or as individual data points, as indicated. Statistical significance between action potential frequencies under control condition compared to substance application was determined by using the Wilcoxon matched-pairs signed rank test (solvent vs. inflammatory soup, solvent vs. TTX). For substance induced changes in time course recordings (Figure 5), significances were calculated using a Friedman test, followed by Dunn’s multiple comparison test (in-cell solvent vs. retigabine, and in-cell solvent vs. NAPQI). Significances comparing different treatmets (Figure 5J) were calculated by a two-way RM ANOVA, followed by Tukey’s post hoch test.

## 3 Results

### 3.1 Loss of responsiveness of DRG neurons towards conventional current pulse injections and inflammtory soup constituents during a 7 days culture period

**FIGURE 1:**
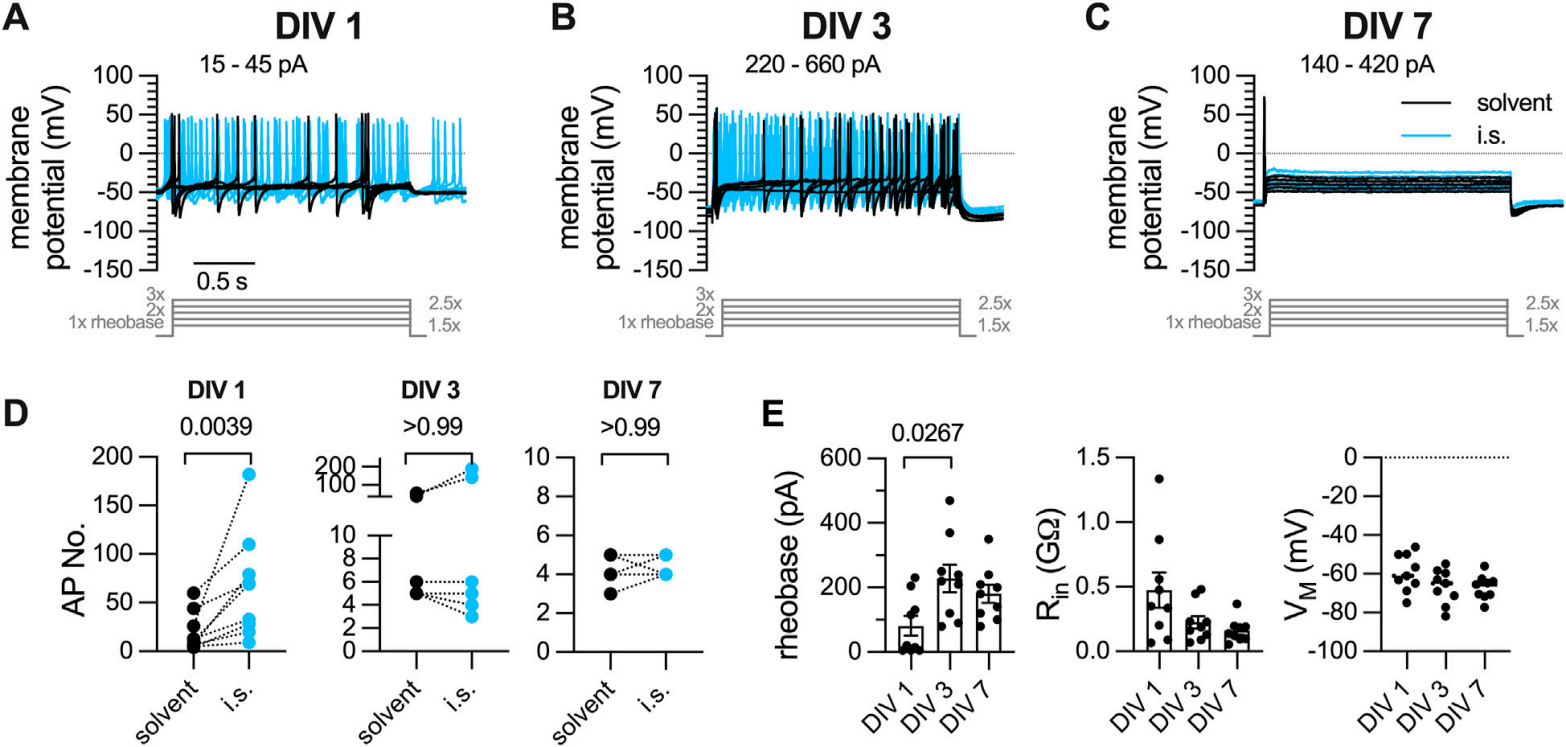
Action potential firing elicited by current pulse injections in DRG neurons after one, three, or 7 days *in vitro* (DIV). DRG neurons were recorded in perforated current-clamp mode; inflammatory soup (i.s.: ADP 1 μ M, ATP 1 μ M, bradykinin 30 nM, histamine 1 μ M, PAR2 agonist 2-Furoyl-LIGRLO-NH2 1 μ M, prostaglandin E2 300 nM, serotonin 300 nM, substance P 10 nM) was present for 30 s before and during triggering of action potentials. **(A)** After determination of the rheobases, five depolarizing current pulses with amplitudes of 1 x, 1.5 x, 2 x, 2.5 x and 3x rheobases (here 15–45 pA) were injected into a DIV 1 neuron for time periods of 2 s each. The traces shown were obtained either in solvent (black traces) or in presence of inflammatory soup components (blue traces). **(B)** Currents were injected as in A (here 220–660 pA) into a DIV 3 neuron. **(C)** shows exemplary voltage traces of a DIV 7 neuron injected with 5 current pulses ranging from 140 to 420 pA. **(D)** statistical analysis of action potential numbers in presence of solvent (0.1% DMSO, black filled circles) and in presence of i.s (blue filled circles) recorded in DIV 1 (left panel, n = 9), DIV 3 (center panel, n = 9), and DIV 7 (right panel, n = 9) neurons (Wilcoxon matched-pairs signed rank test). **(E)** shows the statistical comparison of rheobases (left panel), input resistance ( ${\text{R}}_{in}$ , center panel), and membrane potential ( ${\text{V}}_{M}$ , right panel) of DIV 1, DIV 3, and DIV 7 neurons (Kruskal–Wallis test, followed by Dunn’s multiple comparison *post hoc* test).

Primary cultures of DRG neurons were recorded in perforated current-clamp mode and subjected to a conventional current-injection pulse protocol. After determination of the rheobases for each individual neuron, action potentials were evoked by injection of five current pulses with increasing amplitudes (1 x, 1.5 x, 2 x, 2.5 x, 3 x), each lasting for 2 s. First, this protocol was applied to neurons (n = 9) within 24 h after preparation (DIV 1): 7 out of 9 neurons displayed tonic action potential firing, 2 showed a phasic firing pattern. On average, DIV 1 neurons fired 19.56 ± 18.96 action potentials in presence of solvent (Figure 1A) using an average rheobase of 81.11 ± 91.2 pA (Figure 1E). Thereafter, this protocol was used with neurons after 3 (DIV 3) and 7 days in culture (DIV 7). Two out of 9 neurons showed a tonic firing pattern after 3 days *in vitro* (Figures 1B, D, n = 9) and none of the neurons showed a tonic firing pattern after 7 days in culture (Figures 1C, D, n = 9); on average, DIV 3 neurons fired 14.22 ± 18.46 action potentials in response to an average rheobases of 227.8 ± 128.6 pA (Figures 1D, E) and average rheobases of 180 ± 83.22 pA (Figure 1E) triggered 4.3 ± 0.7 action potentials (Figure 1D) in DIV 7 neurons. Values of input resistance and resting membrane potential did not change during the 7 days culture period (Figure 1E).

A solution mimicking the so-called inflammatory soup (i.s.; Basbaum et al. (2009)) consisting of ADP (1 μ M), ATP (1 μ M), bradykinin (30 nM), histamine (1 μ M), the PAR2 agonist 2-fuoryl-LIGRLO-amide (1 μ M), prostaglandin ${\text{E}}_{2}$ (300 nM), serotonin (300 nM), and substance P (10 nM) was applied for 30 s before a second set of action potentials was triggered by the same conventional current-injection protocol (Figure 1A). This inflammatory soup increased action potential numbers in DIV 1 (Figures 1A, D, left panel), but neither in DIV 3 (Figures 1B, D, center panel) nor in DIV 7 (Figures 1C, D, right panel) DRG neurons.

### 3.2 ${Ca}^{2+}$ responses of DRG neurons are maintained during a 7-day culture period

**FIGURE 2:**
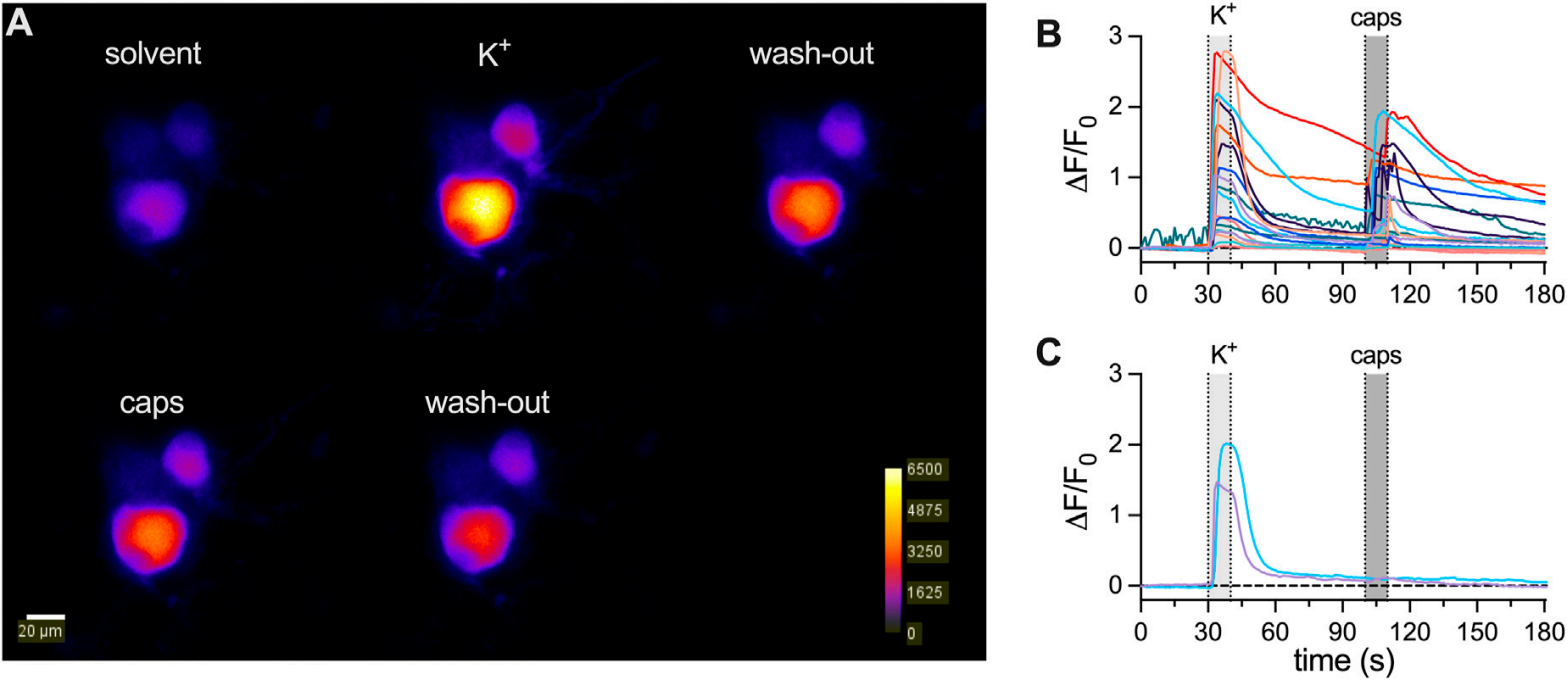
${Ca}^{2+}$ responses of DRG neurons produce ${\text{Ca}}^{2+}$ signals after 7 days in culture. Primary cultures of DRG neurons were seeded onto glass-bottom dishes. The cells were virally transduced 1 day after dissociation with hSYN-jGCamP8s. **(A)** shows representative pseudo color images of a cell population expressing jGCamP8s in presence of solvent (top left panel), 23 mM ${\text{K}}^{+}$ ( ${\text{K}}^{+}$ , top center panel), after wash-out with solvent (wash-out, top right panel), in presence of 300 nM capsaicin (caps, bottom left panel), followed by a second wash-out (wash-out, bottom center panel). Fluorescence intensity is indicated in arbitrary units (A.U.; bottom right). **(B)** shows time courses of jGCamP8s fluorescence changes ( $\Delta {F/F}_{0}$ ) of cells responding to 23 mM ${\text{K}}^{+}$ and 300 nM capsaicin (n = 18) in 4 fields of view. **(C)** shows the time course of jGCamP8s fluorescence changes in cells responding to 23 mM ${\text{K}}^{+}$ , but not to capsaicin (n = 2). ${\text{K}}^{+}$ (23 mM) and capsaicin (300 nM, caps) were applied as indicated.

To reveal whether the apparent decline in membrane excitability might extend to other archetypal responses, ${\text{Ca}}^{2+}$ transients were determined in DIV7 DRG neurons. Cells were transduced with an AAV harboring jGCamP8s under the human synapsin (hSYN) promoter, leading to expression of the construct in neurons only. After 1 week in culture, cells were exposed first to a solution containing 23 mM ${\text{K}}^{+}$ and then to 300 nM capsaicin, both for time periods of 20 s separated by a 4-min wash-out period (Figure 2A). Out of 20 neurons distributed within four fields of view, all cells displayed a rise in cytosolic ${\text{Ca}}^{2+}$ when exposed to 23 mM ${\text{K}}^{+}$ solution, and 18 responded to capsaicin as well (Figures 2B, C).

### 3.3 Action potential firing of DRG neurons in response to white noise stimulation and inflammatory soup constituents is maintained during a 7-day culture period

**FIGURE 3:**
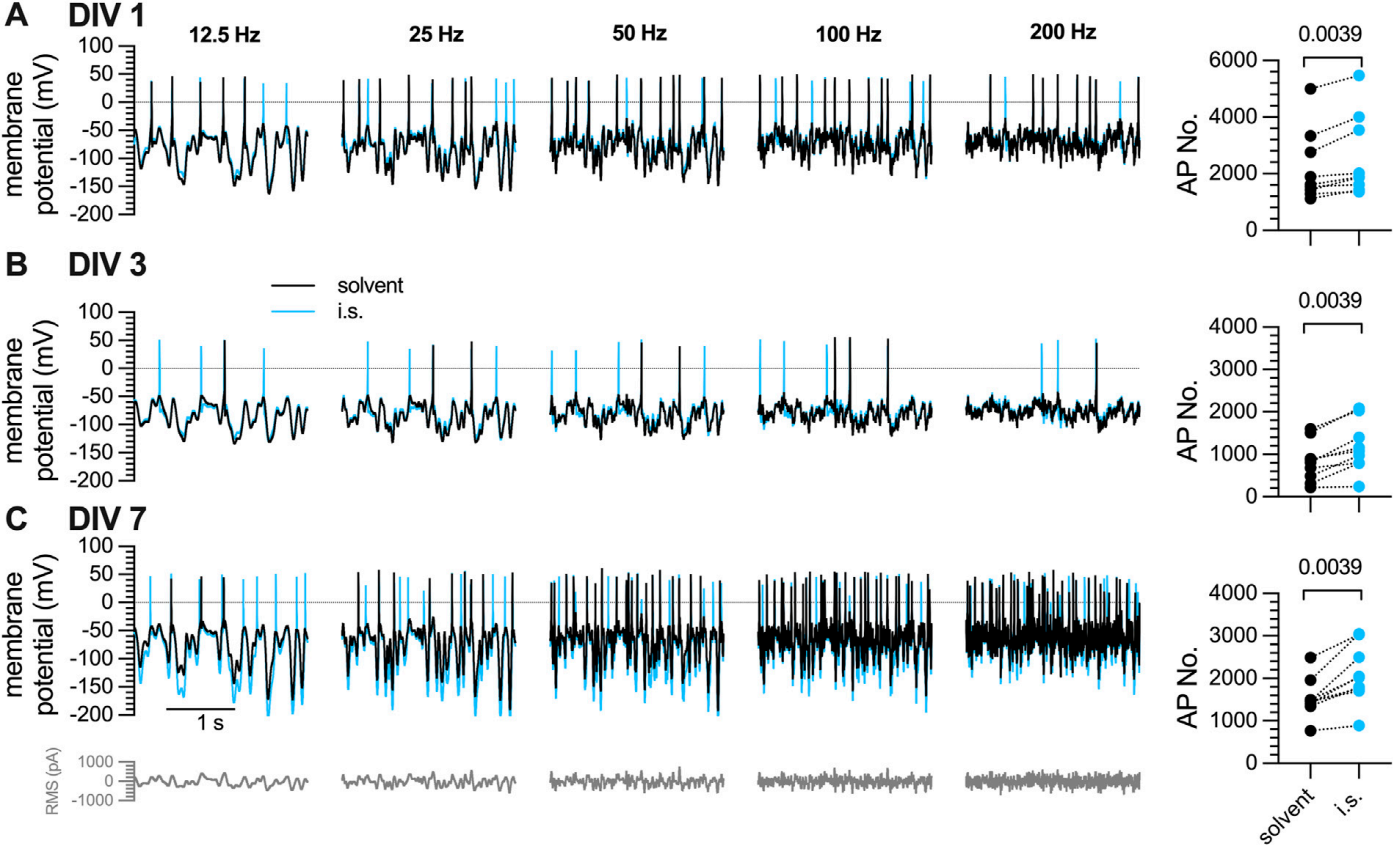
Action potential firing in response to white noise stimuli in DRG neurons after one, three, or 7 days *in vitro* (DIV). The same set of DRG neurons as in Figure 1 were subjected to white noise currents with RMS amplitudes of 50 pA–350 pA in 50 pA increments and their frequencies ranged from 12.5 Hz, 25 Hz, 50 Hz, 100 Hz, to 200 Hz, as indicated. [**(A–C)**, left panels] The traces shown were obtained with a current amplitude of 200 pA either in solvent (black traces) or in presence of inflammatory soup components (blue traces) (A) after 1 day *in vitro* (DIV 1), **(B)** after 3 days in culture (DIV 3), and **(C)** after 7 days *in vitro* (DIV 7). [**(A–C)**, right panel] shows the statistical analysis of all action potentials triggered in **(A)** 1 DIV, **(B)** DIV 3, and **(C)** 7 DIV neurons by white noise stimulation in presence of solvent (black circles; n = 9) vs. inflammatory soup (i.s., blue circles; n = 9); the level of statistical significance evaluated by a Wilcoxon matched-pairs signed rank test is indicated above the symbols.

The discrepancy between maintained ${\text{Ca}}^{2+}$ responses and the apparent loss of action potential firing after a 7-day culture period triggered a search for alternative stimulation paradigms. Fifty years ago, Gaussian white noise stimulation has been reported to trigger action potential firing in squid giant axons (Guttman et al., 1974) and *Aplysia* neurons (Bryant and Segundo, 1976), but this strategy was no longer pursued thereafter. Accordingly, the neurons exposed to rectangular current injections after one, 3 or 7 days *in vitro* (Figure 1), were rechallenged by Gaussian white noise stimuli with frequencies ranging from 12.5 Hz to 200 Hz and RMS stimulus amplitudes of 50 pA–350 pA in 50 pA increments (Figure 3). This protocol triggered tens to hundreds of action potentials, and exposure to the same i.s as used in Figure 1 increased numbers of action potential triggered by Gaussian white noise irrespective of the preceding culturing period (Figure 3).

### 3.4 Semi-automated detection of action potentials triggered by Gaussian white noise stimuli

**FIGURE 4:**
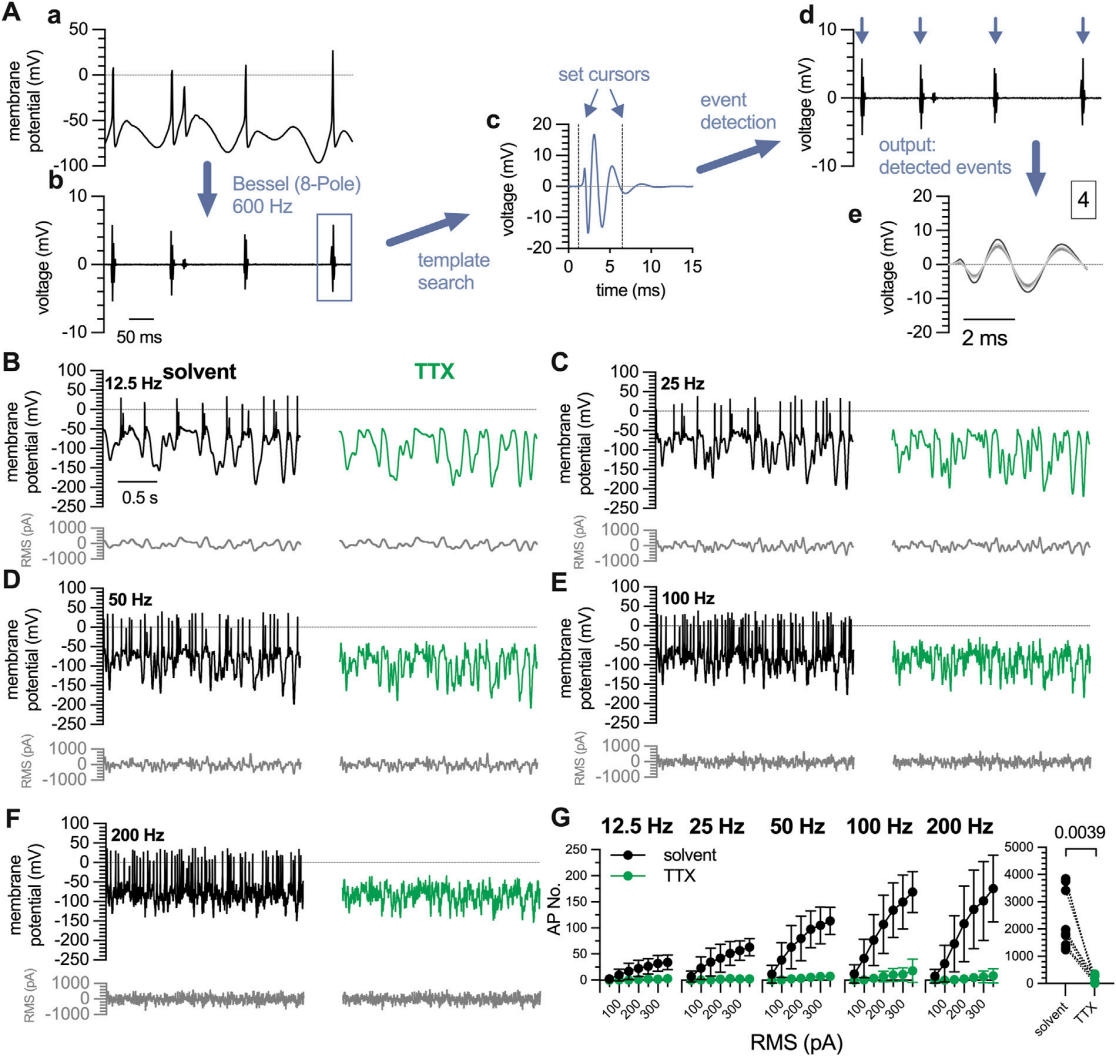
Characterization of action potentials triggered by white noise stimulation in 7 DIV DRG neurons. A complete perforated current-clamp recording consists of 35 traces (10 s each) recorded at filter frequencies of 12.5 Hz, 25 Hz, 50 Hz, 100 Hz, and 200 Hz with RMS amplitudes ranging from 50 pA to 350 pA, in 50 pA increments. **(A)** Displays the quantification of action potentials triggered by white noise stimulation. (a) For clarity, only 0.5 s of a representative 10 s trace recorded at 12.5 Hz and 200 pA are shown. (b) Traces are filtered using a Bessel (8-pole) high-pass filter at a cut-off frequency of 600 Hz. (c) Then a template search is conducted in Clampfit 10.7 using the template shown in (c). The cursors defining the beginning and the end of the template can be adjusted manually as indicated by the blue arrows. (d) Using this template, events are detected automatically, indicated by blue arrows, and are available for visual inspection. (e) The output window displays all detected events in expanded time scale and indicates the total number of events (here 4). **(B–F)** show exemplary traces obtained in the very same neuron using a white noise stimulation protocol as indicated in grey below each trace. The white noise currents had RMS amplitudes of 50 pA–350 pA with 50 pA increments and their frequencies ranged from **(C)** 12.5 Hz, **(D)** 25 Hz, **(E)** 50 Hz, **(F)** 100 Hz, to **(G)** 200 Hz. The traces shown were obtained with a current amplitude of 200 pA either in solvent (black traces) or in presence of tetrodotoxin (TTX, 1 μ M, green traces). **(G)** shows average numbers of all action potentials (AP No.) triggered by each of the white noise stimulation frequencies and amplitudes in presence of solvent (black circles; n = 9) or TTX (green circles; n = 9); the level of statistical significance is evaluated by a Wilcoxon matched-pairs signed rank test and indicated above the symbols.

The Gaussian white noise stimulation protocol triggered an average of 2,285 ± 1,074 action potentials in DIV 7 neurons (Figure 4G, solvent), a number by far too high for experimenter driven single event evaluation. Consequently, some automated event detection was required to economize analyses of white noise evoked neuronal firing. To this end, original traces obtained during white noise stimulation (Figure 4Aa) were filtered using a Bessel (8-pole) high-pass filter at a frequency of 600 Hz (Figure 4Ab). Subsequently, the built-in “template search function” of Clampfit 10.7 was utilized (Figure 4Ac), and on the basis of the resulting template action potentials were detected (Figure 4Ad); the numbers of these were reported in the output window (Figure 4Ae).

To probe the validity of the Gaussian white noise trigger and this semi-automated detection method, action potentials were first recorded under standard conditions and thereafter in presence of tetrodotoxin (TTX, 1 μ M), which blocks voltage-gated ${Na}^{+}$ channels (Alexander et al., 2023). TTX curtailed the occurrence of action potentials at all stimulation frequencies (Figures 4B–F) and RMS stimulus amplitudes (Figure 4G). In TTX, the total number of action potentials was reduced to 116.1 ± 137.4 (Figure 4G). Three out of 9 cells recorded, fired more than 250 action potentials in presence of TTX, whereas in the remaining 6 cells, 6 to 69 action potentials were detected during exposure to TTX.

### 3.5 White noise stimulation but not conventional pulse protocols permit the detection of analgesic drug actions

**FIGURE 5:**
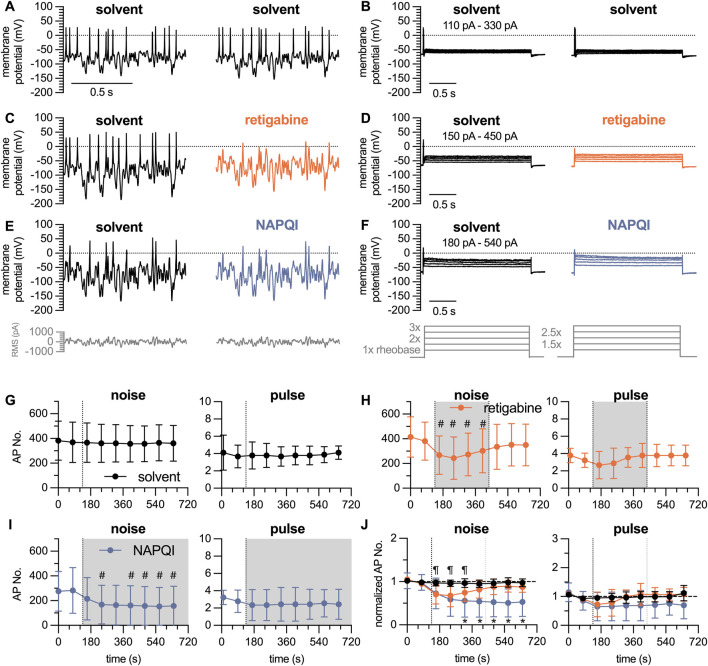
Time course of the effects of analgesics on action potential firing in DIV 7 DRG neurons analyzed by white noise stimulation vs. current pulses. The white noise protocol (noise) consisted of stimulation frequencies ranging from 12.5 Hz, 25 Hz, 50 Hz, 100 Hz and 200 Hz with RMS amplitudes of 100 pA–300 pA in 50 pA increments; each individual stimulus lasted for 2.5 s to give a total white noise stimulation period of approximately 1 minute. This was followed by a 5-step current pulse protocol (pulse) ranging from 1 x to 3 x rheobases with 0.5 x increments, each step lasting for a total of 3 s. After establishing baseline values during a 150 s period (two noise protocols and two pulse protocols) in solvent (DMSO 0.1%), solvent or retigabine were present for 300 s followed by a 300 s wash out period in solvent; alternatively, NAPQI was present for 600 s **(A–F)** show representative traces in presence of **(A, B)** solvent only, **(C, D)** solvent followed by retigabine (10 μ M), and **(E, F)** solvent followed by NAPQI (3 μ M), respectively. **(A, C, E)** display responses to white noise stimulation with a frequency of 100 Hz and an RMS amplitude of 200 pA as indicated below (grey traces). **(B, D, F)** display responses to the 5-step pulse protocol recoded in the very same cells as **(A, C, E)**; the current injection protocol is indicated below (grey traces). **(G–I)** Display the time course of action potentials in response to white noise stimulation (noise; left panels) and current pulses (pulse; right panels) in neurons exposed to **(G)** solvent (DMSO 0.1%, n = 9), **(H)** retigabine (10 μ M, n = 9) # p = 0.0033 (at 160 s), # p = 0.0001 (at 240 s), # p = 0.0001 (at 320 s), # p = 0.0039 (at 400 s) vs. solvent at time 0 s, and **(I)** NAPQI (3 μ M, n = 9); # p = 0.315 (at 24 s), # p = 0.0156 (at 400 s), # p = 0.0017 (at 480 s), # p = 0.0001 (at 560 s), and # p = 0.0006 (at 640 s) vs. solvent at time 0 s (Friedman test, followed by Dunn’s multiple comparisons *post hoc* test). **(J)** shows normalized amplitude values of all cells shown in **(G–I)**. Onset of substance application is marked by the black dotted line (135 s), wash-out of retigabine is indicated by the grey dotted line (435 s). Retigabine vs. solvent: ¶ p = 0.0175 (at 180s), ¶ p = 0.0268 (at 240 s), ¶ p = 0.0333 (at 320 s); NAPQI vs. solvent: ∗ p = 0.0278 (at 320 s), ∗ p = 0.0235 (at 400 s), ∗ p = 0.0121 (at 480 s), ∗ p = 0.0055 (at 560 s), and ∗ p = 0.0098 (at 640 s) (two-way RM ANOVA followed by Tukey’s multiple comparison *post hoc* test).

The Gaussian white noise stimulation presented above consisted of 35 individual stimuli lasting for 10 s each, which summed up to a total duration of 350 s. Thus, in time course experiments one could obtain only one value per 6 min. To enable the detection of time-dependent effects of drugs on action potential firing, a modified white noise protocol was generated: this covered stimulation frequencies of 12.5 Hz, 25 Hz, 50 Hz, 100 Hz, and 200 Hz and RMS amplitudes reduced to values from 100 pA to 300 pA in 50 pA increments. This resulted in 25 individual stimuli with a duration of 2.5 s each, summing up to a total stimulation time of 62.5 s. Alternating with this shortened white noise protocol, a conventional pulse protocol was used. This consisted of 5 individual current injections lasting for 3 s each, ranging from 1 x, 1.5 x, 2 x, 2.5 x to 3 x rheobases; this gave a total stimulus time of 15 s (Figures 5A, B). Using this approach, rather stable numbers of action potentials can be triggered over a time period of 10 min in presence of solvent (DMSO 0.1%, Figure 5G). To test the sensitivity of this approach towards inhibitory actions of drugs, the ${\text{K}}_{V}$ 7 channel activator retigabine (10 μ M) was employed, as this agent has been found to interfere with sensory neuron excitability (Passmore et al., 2003). When retigabine was present for periods of 5 min, numbers of action potentials triggered by Gaussian white noise stimulation were reduced (Figures 5C, H), but action potentials elicited by conventional current-injection protocols in the very same neurons remained unaltered (Figures 5D, H).

The paracetamol metabolite N-acetyl-p-benzoquinone imine (NAPQI) has been found to cause a slowly developing decrease in the number of action potentials fired by DIV 1 DRG neurons via a time-dependent irreversible increase in currents through ${\text{K}}_{V}$ 7 channels (Ray et al., 2019). Here, NAPQI (3 μ M) was applied for periods of 600 s and action potentials triggered by the shortened white noise protocol were reduced (Figures 5E, I), whereas those elicited by the conventional pulse protocol were unaffected (Figures 5F, I). Numbers of action potentials whether evoked by Gaussian white noise stimulation or conventional pulse protocols varied considerably between single neurons. Hence, to better resolve time-dependent drug actions, numbers of action potential triggered by a single set of white noise stimuli and current pulses, respectively, were normalized to an average of the first two in a series of 9 stimuli (Figure 5J). With white noise stimulation, there was virtually no variation of normalized data over a time period of 10 min. The results obtained with current injections, however, displayed considerable variability. Moreover, retigabine as well as NAPQI caused significant reductions in such normalized analyses of action potentials triggered by white noise stimulation, but failed to do so with action potentials evoked by conventional pulse protocols (Figure 5J).

## 4 Discussion

Effects of various agents on membrane excitability in neurons can be studied by current clamp recordings. Traditionally, action potential firing is triggered by protocols that inject rectangular current pulses that stay constant over time periods of hundreds of milliseconds or even a few seconds (Hodgkin, 1948; Spindler et al., 1999; Zaika et al., 2006; Dominguez-Rodriguez et al., 2017). This, however, is quite different from the *in vivo* situation in the nervous system where a single neuron can receive multiple input signals via synaptic connections from other neurons, i.e., excitatory and inhibitory postsynaptic potentials which are then spatially and temporally integrated to generate an output response (Eccles et al., 1966; Chavas and Marty, 2003). Gaussian white noise generates random alternating depolarizing and hyperpolarizing stimuli in the range of a few milliseconds and thereby aims to mimic these physiological conditions. Excitation of nociceptors occurs through stimuli that impinge as mechanical, thermal and/or chemical signals on a group of approximately 30 different transducers. These signals accumulate and dissipate in the vicinity of nociceptors in a random manner and thus lead to a random pattern of generator potentials that are then transformed into action potentials (Reichling and Levine, 1999; Gold and Gebhart, 2010). Again, this random mode of excitation is likely to be mimicked better by Gaussian white noise rather than rectangular current pulses. Additionally, a prolonged depolarizing stimulus leads to voltage-dependent inactivation of voltage-gated sodium channels (Goldman and Schauf, 1972), and their recovery from inactivation is dependent on the extent of a subsequent hyperpolarization (Kuo and Bean, 1994). Therefore, the proportion of inactivated sodium channels must be expected to be larger during injection of current pulses as compared to a white noise stimulus that includes intermittent hyperpolarizations. Based on these considerations, the present experiments employed Gaussian white noise to trigger action potentials in DRG neurons.

Dissociated cultures of DRGs have been used to analyze mechanisms and consequences of axotomy and were found to express injury related proteins in a time-dependent manner, with low levels immediately after dissociation and a rise during the subsequent days in culture (Levin et al., 2017; Cronin et al., 2022). As axonal injury can lead to the development of neuropathic pain (Testa et al., 2024), one would expect that dissociated DRG neurons experience an increase in excitability during extended periods of culturing in parallel with the appearance of axotomy-induced proteins. However, the present data provide evidence to the opposite: DIV 1 neurons fired 19 action potentials during 2 s current injections with amplitudes corresponding to 1 x, 1.5 x, 2 x, 2.5 x, 3 x rheobases and an average rheobases of 81 pA; DIV 3 neurons fired 14 action potentials with an average rheobases of 227 pA, DIV 7 neurons fired 4 action potentials with an average rheobases of 180 pA. Thus, membrane excitability of sensory neurons progressively decreases as they are kept in culture for several days to 1 week.

The tendency of DRG neurons to fire action potentials in response to appropriate stimuli is not fixed, but rather influenced by various parameters such as, for instance, temperature (Jia et al., 2012). More relevant in terms of pathology and therapy is the impact of algogenic and/or analgesic agents on the excitability of DRG neurons. In principle, this excitability is enhanced by toxicants that cause peripheral neuropathy (Li et al., 2021) or mediators of inflammation (Basbaum et al., 2009) and reduced by analgesic drugs (Yang et al., 2009; Ray et al., 2019). Here, numbers of action potentials fired in response to conventional current pulse injections were increased by a mixture of inflammatory mediators in DIV 1, but neither DIV 3 nor DIV 7 neurons. Hence, after 1 day in culture, DRG neurons not only become more resistant towards stimuli that are usually well suited to trigger action potentials, they also lose their capability to react to constituents of the so-called inflammatory soup (Basbaum et al., 2009). Moreover, when neurons can fire only one action potential per current pulse injection, any analgesic drug is rather unlikely to be able to exert inhibitory actions on the firing frequency. Thus, DIV 3 and DIV 7 cultures have lost their potential to reliably display drug-induced changes in excitability when stimulated by injection of conventional current pulses.

As alternative method of triggering action potentials in DRG neurons in primary culture, white noise stimulation was employed. Application of the generated waveforms elicited action potentials in a highly reliable manner over extended periods of time. Filter frequencies and RMS amplitudes of the white noise stimuli were changed to evaluate effects of these parameters on neuronal excitability. Higher frequencies and rising RMS amplitudes led to greater numbers of action potentials. Nevertheless, the general firing pattern remained comparable across different filter frequencies in the same cell (Figure 4). Moreover, numbers of action potentials fired in response to white noise stimulation remained stable over periods of 10 min or even more (Figure 5G), and after normalization, the variability between neurons was limited and by far less pronounced when compared to the results obtained with current pulse injection (Figure 5J). Thus, Gaussian white noise appears to be a very reliable trigger of action potential firing in DRG neurons.

Despite the reliability of white noise stimulation, the resulting firing of action potentials readily responded to the presence of agents that are expected to modulate membrane excitability of DRG neurons. This was not only true for the inflammatory mediators ADP, ATP, bradykinin, histamine, prostaglandin ${\text{E}}_{2}$ , serotonin, and substance P as well as the PAR2 agonist 2-fuoryl-LIGRLO-amide which are known to increase DRG neuron excitability through activation of G protein-coupled receptors (Salzer et al., 2019). The reactivity of the firing pattern evoked by Gaussian white noise was also documented by the inhibitory actions of retigabine and the paracetamol metabolite NAPQI which reduce sensory neuron excitability through an enhancement of currents through ${\text{K}}_{V}$ 7 channels (Passmore et al., 2003; Ray et al., 2019). This proved that DRG neurons after 3 and 7 days in culture did retain their potential to experience a modulation of excitability by agents that exert algogenic or analgesic effects at the level of nociceptors.

Taken together, the experiments shown above establish Gaussian white noise as reliable method of eliciting action potential firing in sensory neurons that appears to be even more similar to the physiological situation as current pulse injection. By this stimulation paradigm, the apparent loss of excitability of DRG neurons due to extended culturing periods as observed with current pulse injection can be overcome and at the same time the reactivity of action potential firing towards algogenic and analgesic agents is preserved.

## Data Availability

The raw data supporting the conclusions of this article will be made available by the authors, without undue reservation.

## Abbreviations

AAV, adeno-associated viral; DIV, days *in vitro*; DRG, dorsal root ganglion; FBS-hi, fetal bovine serum - heat inactivated; i. s., inflammatory soup; NAPQI, n-acetyl-p-benzoquinone imine; MES, 2-(N-morpholino)ethanesulfonic acid; RMS, root mean square; TTX, tetrodotoxin.
