# Supplementary material for: Gaussian white noise stimulation as an alternative method to excite sensory neurons
Source: Front Pharmacol. 2025 Apr 22;16:1561905. doi: 10.3389/fphar.2025.1561905 (PMC12053156; doi:10.3389/fphar.2025.1561905)
Supplement: Supplementary file 1 [file DataSheet1.pdf]

```
function generateNoiseStimulusSequence(fs, timePerSweep, numFilters,
filterCutoff, SDs, normalize)
% GENERATESTIMULUSFILE Generates a Axon atf file containing a white
noise sequence
% inputs:
% fs ... sampling frequency (Hz)
% timePerSweep ... sequence duration (s)
% numFilters ... number of lowpass filters starting from
'filterCutoff'.
% Each cutoff frequency is reduced by factor 2 from the previous
(e.g.
% filterCutoff = 200 Hz, numFilters = 5 -> filter cutoffs will be
200, 100,
% 50, 25, 12.5 Hz
% filterCutoff ... starting cutoff for bessel filter
% SDs ... array of noise amplitudes to use (in nA)
% normalize ... [0 or 1] if 1 scale filtered data after filtering,
so that
% noise amplitutde is equal to the SD set. if 0 do not reascale data
%
% example: generateNoiseStimulusSequence(20e3, 2, 5, 200, [0.100,
0.150, 0.200, 0.250, 0.300], 1)
% generates a 2 s stimulus, with 5 different cutoff frequencies
starting from 200 Hz
% (reduced by factor 2 for each consecutive filter), with SDs of
100, 150 ... 300 pA
% at as sampling freq. of 20 kHz


numAmplitudes = length(SDs);

%add 0.25 s of 0 nA signal to the start and end of each sweep
padding = 0.25;
pointsPerSweep = fs * timePerSweep + 2 * padding * fs; %Number of
sampling points per sweep

numSweeps = numFilters * numAmplitudes;

%initialize random number generator
rng(0);

% generate gaussian noise with mu = 0, SD = 1
noiseTrace = randn(timePerSweep * fs , 1);

% initialize sweep to 0
sweep = zeros(pointsPerSweep, 1);

% set sweep between the 0 padding region to noiseTrace
sweep(padding * fs : padding * fs + timePerSweep * fs - 1) =
noiseTrace;

% initialize output array as 0
data = zeros(length(sweep), numSweeps);
```

```
% apply bessel filter
for i = 1:numFilters
    % get filter coefficients
    [b, a] = besself(4, filterCutoff * 2 * pi, 'low');
    [bz, az] = impinvar(b,a,fs);
    % apply zero phase filter
    data(:,i) = filtfilt(bz,az,sweep);
    % calculate next filter cutoff frequency
    filterCutoff = filterCutoff / 2;
end
% normalize filtered data
if normalize == 1
    data = data-mean(data);
    data = data./std(data);
end

rawData = data;

% scale the SD of the filtered noise by the user input
for i = 1:numAmplitudes
    data(:, 1 + (i-1) *numFilters : (i-1) * numFilters + numFilters)
= rawData(:, 1 : numFilters) * SDs(i);
end

sweepInterval = timePerSweep + padding * 2;

% open Axon ATF File
fileID = fopen('stim_short.atf', 'w');

% set file type to ATF 1.0
fprintf(fileID,'ATF\t1.0\n');

% set number of sweeps in file
fprintf(fileID,'8\t%d\n',numSweeps + 1);

% set to Aquistion mode to "Episodic"
fprintf(fileID,'"AcquisitionMode=Episodic Stimulation\n"');
fprintf(fileID,'"Comment="\n');

% set scaling factors for displaying in clampFit
fprintf(fileID,'"YTop=500"\n');
fprintf(fileID,'"YBottom=-500"\n');

% set sampling interval in µs
fprintf(fileID,'"SyncTimeUnits=%d"\n',(1/fs)*1e6);

% set the starting times and intervals for each sweep
fprintf(fileID,'"SweepStartTimesMS=');
    for i = 0:numSweeps-2
        fprintf(fileID,'%f,\t', i*sweepInterval);
    end
fprintf(fileID,'%f"\n',(i+1) * sweepInterval);

% set Signal name
```

```
fprintf(fileID,'"SignalsExported=IN 0"\n');

% print Signal headers
fprintf(fileID,'"Signals="\t');
for i =1:numSweeps
    fprintf(fileID,'"IN 0"\t');
end
fprintf(fileID,'\n');
fprintf(fileID,'"Time (s)"\t');
for i = 1:numSweeps
    fprintf(fileID,'"Trace #%d"\t',i);
end
fprintf(fileID,'\n');

% print out signal data, row by row
for i =1:size(data, 1)
    fprintf(fileID, '%f\t', i/fs);
    for j = 0 : numSweeps - 1
        fprintf(fileID, '%f\t',data(i, j+1));
    end
    fprintf(fileID, '%f\n',data(i, j+1));
end

% close file
fclose(fileID);

end
```
